# Molecular Characterization of *Cryptosporidium* spp., *Giardia duodenalis*, *Enterocytozoon bieneusi* and *Escherichia coli* in Dairy Goat Kids with Diarrhea in Partial Regions of Shaanxi Province, China

**DOI:** 10.3390/ani13182922

**Published:** 2023-09-14

**Authors:** Xin Yang, Junwei Wang, Shuang Huang, Junke Song, Yingying Fan, Guanghui Zhao

**Affiliations:** 1 College of Veterinary Medicine, Northwest A&F University, Yangling 712100, China; xinyang@nwafu.edu.cn (X.Y.); wjunwei@nwafu.edu.cn (J.W.); huangshuang7892021@163.com (S.H.); sjk7998@163.com (J.S.); yingyingfan@nwafu.edu.cn (Y.F.); 2 Engineering Research Center of Efficient New Vaccines for Animals, Ministry of Education, Yangling 712100, China; 3 Key Laboratory of Ruminant Disease Prevention and Control (West), Ministry of Agriculture and Rural Affairs, Yangling 712100, China; 4 Engineering Research Center of Efficient New Vaccines for Animals, Universities of Shaanxi Province, Yangling 712100, China

**Keywords:** *Cryptosporidium* spp., *Giardia duodenalis*, *Enterocytozoon bieneusi*, *Escherichia coli*, goat, Shaanxi

## Abstract

**Simple Summary:**

*Cryptosporidium* spp., *Giardia duodenalis*, *Enterocytozoon bieneusi* and *Escherichia coli* are major zoonotic pathogens causing diarrhea in humans and various animals. Knowledge of the distribution and genetic diversity of pathogens can shed a new light on the prevention and control of diseases. This study investigated the colonization frequency and genetic make-up of *Cryptosporidium* spp., *G. duodenalis*, *E. bieneusi* and *E. coli* in dairy goat kids with diarrhea in partial regions of Shaanxi Province. The frequent occurrence of zoonotic species/genotypes/subtypes/pathotypes of these four pathogens in the present study indicated the potential for zoonotic transmission between humans and animals.

**Abstract:**

*Cryptosporidium* spp., *Giardia duodenalis*, *Enterocytozoon bieneusi* and *Escherichia coli* are important diarrheal pathogens threatening the health of humans and various animals. Goats, especially pre-weaned goat kids, that carry these pathogens are important reservoirs related to human infection. In the present study, PCR-based sequencing techniques were applied to characterize *Cryptosporidium* spp., *G. duodenalis*, *E. bieneusi* and *E. coli* in 202 fecal samples of diarrheal kids for Guanzhong dairy goats from five locations in Shaanxi Province. The positive rates of *Cryptosporidium* spp., *G. duodenalis*, *E. bieneusi* and *E. coli* were 37.6% (76/202), 16.3% (33/202), 55.4% (112/202) and 78.7% (159/202) in these goat kids, respectively. Co-infection of two to four pathogens was found in 114 of 202 fecal samples. Significant differences (*p* < 0.001) in the positive rates of *Cryptosporidium* spp. and *G. duodenalis* were found among locations and age groups. Furthermore, two *Cryptosporidium* species (*C. parvum* and *C. xiaoi*), two *G. duodenalis* assemblages (E and A), nine *E. bieneusi* genotypes (CHG3, CHG1, BEB6, CHG5, CHG2, SX1, CHG28, COS-II and CD6) and two *E. coli* pathotypes (EPEC and EHEC) were identified. As for *Cryptosporidium*, two (IIdA19G1 and IIdA19G2) and two (XXIIIa and XXIIIg) subtypes were recognized in samples positive for *C. parvum* and *C. xiaoi*, respectively. A phylogenetic analysis based on the ITS locus of *E. bieneusi* indicated that all nine genotypes of *E. bieneusi* identified in this study belonged to the group 2. Four virulence factors (*ehxA*, *eae*, *stx2* and *stx1*) of EPEC and EHEC were found in *E. coli* strains. Collectively, this study explored the colonization frequency of *Cryptosporidium* spp., *G. duodenalis*, *E. bieneusi* and *E. coli* in diarrheal kids of Guanzhong dairy goats in Shaanxi Province and expanded our understanding of the genetic composition and zoonotic potential of these pathogens in goats.

## 1. Introduction

*Cryptosporidium* spp., *Giardia duodenalis*, *Enterocytozoon bieneusi* and *Escherichia coli* are important zoonotic pathogens closely related with the occurrence of diarrhea, significantly endangering the health of humans and various animals [1,2,3,4,5]. These pathogens can be transmitted between humans and animals via several routes, e.g., consumption of contaminated food/water and direct contact with infected persons/animals [6,7,8,9]. Although *Cryptosporidium* spp., *G. duodenalis*, *E. bieneusi* and *E. coli* are usually reported as opportunistic pathogens, they have caused hundreds of outbreaks of diarrhea in humans and animals, resulting in considerable economic losses for human health and the animal-breeding industry [10,11,12,13,14]. In addition to the public health significance, infections of these pathogens can also cause growth retardation in children and immune-compromised individuals [15,16,17,18].

Advances in the occurrence and molecular make-up of pathogens shed a novel light on the prevention and treatment of diseases. Recently, over 40 species and 100 genotypes of *Cryptosporidium* have been found in humans and various animals based on a molecular analysis targeting the small subunit ribosomal RNA gene (*SSU rRNA*) [19]. Anywhere from one to four *Cryptosporidium* species/genotypes have been recognized in one host species, e.g., the occurrence of *Cryptosporidium hominis* and *C. parvum* in humans, *C. canis* in dogs, and *C. parvum*, *C. bovis*, *C. ryanae* and *C. andersoni* in cattle [20]. Among those identified species and genotypes, some of them, e.g., *C. parvum*, *C. hominis*, *C. meleagridis*, *C. felis*, *C. canis* and *C. ubiquitum*, are zoonotic, threatening the health of both humans and animals [20]. Further subtyping analyses targeting the 60-kDa glycoprotein gene (*gp60*) contribute to the understanding of transmission and tracing studies of *Cryptosporidium* spp. [10,21,22,23]. Based on the genetic diversity of the *beta-giardin* gene (*bg*), *glutamate dehydrogenase* gene (*gdh*) and *triose phosphate isomerase* gene (*tpi*), a total of eight assemblages (A–H) were identified in *G. duodenalis* [3]. Of them, a wide host range was found for assemblages A and B, which can infect humans and various mammals. Assemblages C and D are mainly found in canines. Assemblage E is commonly recognized in artiodactyls. Assemblages F, G and H are frequently identified in felines, rodents and marine mammals, respectively [3,10,24]. As for *E. bieneusi*, 11 genetic groups (groups 1–11) with divergent host specificity have been identified based on PCR sequencing of the internal transcribed spacer (ITS) gene [6,25]. Most genotypes in the group 1, e.g., Type IV, EbpC, D and Peru6, are frequently identified in humans and various animals, indicating the zoonotic importance of this group [6]. Although genotypes from the group 2 were reported to be ruminant-specific at the beginning, an expanding host range for more and more genotypes (e.g., BEB4, BEB6 and I) reflects increasing zoonotic potential within this group [12,26,27]. Host specificities of genotypes are frequently found in the groups 3–11, indicated by the unique occurrence of PtEb VIII and WL6 in cats and rodents, respectively [6]. *Escherichia coli* is one of the most common bacteria in animals and humans with important significance, especially for pathogenic *E. coli*. Pathogenic *E. coli* can produce toxins and is classified into two groups, namely intestinal (IPEC) and extra-intestinal (ExPEC) pathogenic *E. coli*, based on divergent virulence factors (e.g., toxins and adhesin) [28]. Further, IPEC can be sub-classified into enteropathogenic (EPEC), enterotoxigenic (ETEC), enteroaggregative (EAEC), enteroinvasive (EIEC) and enterohemorrhagic (EHEC) *E. coli* [29], causing diarrhea, inflammation or even death in infected children and young animals [30].

As one of the most important livestock in China, goats, which can carry *Cryptosporidium* spp., *G. duodenalis*, *E. bieneusi* and *E. coli*, are important reservoirs related to human infections, threatening the health of humans and development of the breeding industry [31,32,33,34]. The Guanzhong dairy goat is a famous and excellent breed of dairy goat in China and is mainly distributed in Shaanxi Province. However, little is known of the occurrence of these pathogens in Guanzhong dairy goats. This study investigates the positive rates and genetic composition of *Cryptosporidium* spp., *G. duodenalis*, *E. bieneusi* and *E. coli* in diarrheal kids of Guanzhong dairy goats, and the results expand our understanding of the distribution and zoonotic potential of these diarrhea-related pathogens in goats.

## 2. Materials and Methods

### 2.1. Samples

**Figure 1:**
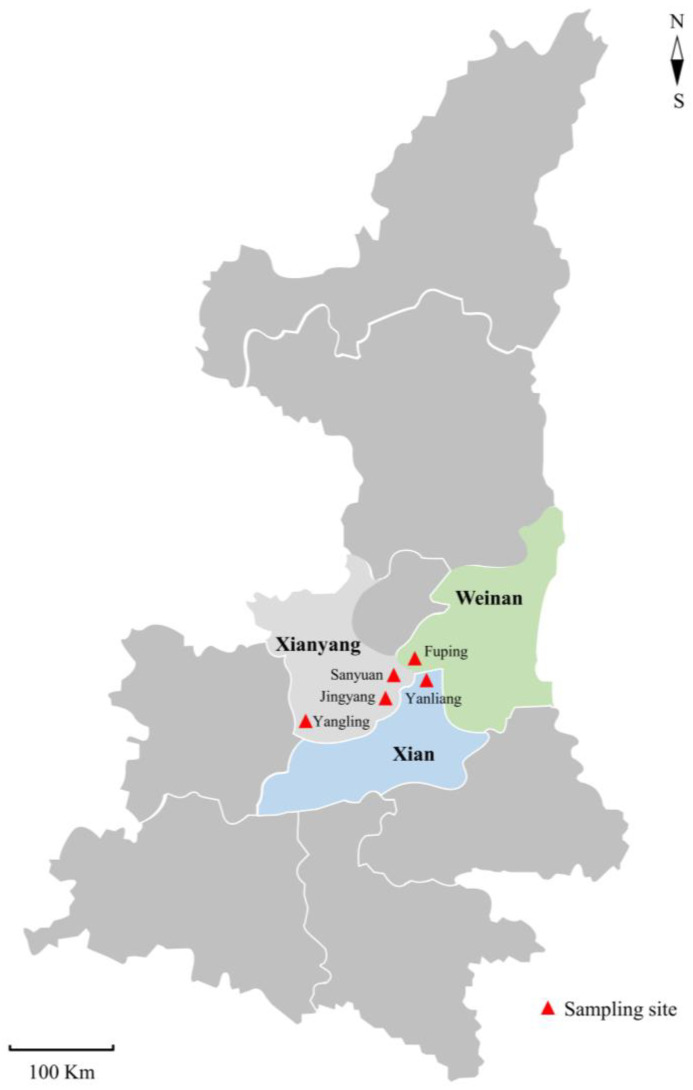
Geographical distribution of sampling sites of dairy goat kids with diarrhea in partial regions of Shaanxi Province in the present study.

From February 2022 to May 2023, a total of 202 fecal samples were collected from diarrheal Guanzhong dairy goat kids in five locations of Shaanxi Province (Figure 1), containing 47, 102 and 53 fecal samples from goat kids aged <2 weeks, 2–4 weeks and 4–12 weeks, respectively. All samples were directly collected from the rectum of goat kids using sterile cotton swabs (HUNAUT, Qingdao, Shandong, China), placed into separate 50 mL centrifuge tubes (Thermo Fisher Scientific, Waltham, MA, USA), marked with sampling date, city name, location and age, and transported to the Parasitology Lab of Northwest A&F University for examination under cool conditions as soon as possible.

### 2.2. Genomic DNA Extraction

Genomic DNA was isolated from fecal samples using an E.Z.N.A. Stool DNA kit (Omega, Norcross, GA, USA) as per the manufacturer’s instructions. All gDNA samples were kept at −20 °C until use.

### 2.3. Detection, Genotyping and Subtyping of Cryptosporidium spp., G. duodenalis and E. bieneusi

A nested PCR-based sequencing technique targeting the *SSU rRNA* gene (~830 bp) was used to investigate the positive rates and species compositions of *Cryptosporidium* spp. in goat kids, as previously reported [35]. Then, *C. parvum* and *C. xiaoi* identified in the present study were subtyped using two nested PCR-based sequencing techniques of the *gp60* gene [21,36].

The positive rates and assemblages of *G. duodenalis* were investigated by applying three nested PCR-based sequencing assays targeting the *tpi* gene (~530 bp) [37], *bg* gene (~511 bp) [38] and *gdh* gene (~599 bp) [39].

A nested PCR-based sequencing technique targeting the ITS gene locus (~392 bp) was used to determine the positive rates and genotypes of *E. bieneusi* in fecal samples, as previously reported [40].

### 2.4. Sequence Analysis

All positive secondary PCR products were sent to Sangon Biotech (Shanghai, China) for sequencing in both directions. The obtained sequences were assembled, edited and aligned using ChromasPro V1.33 (www.technelysium.com.au/ChromasPro.html (accessed on 15 June 2023)), BioEdit V7.04 (www.mbio.ncsu.edu/BioEdit/bioedit.html (accessed on 15 June 2023)) and ClustalX V2.1 (www.clustal.org/ (accessed on 15 June 2023)), respectively. To assess the relationships of *Cryptosporidium* spp., *G. duodenalis* and *E. bieneusi* found in the present study, three phylogenetic trees were constructed with the maximum likelihood (ML) method, a general time-reversible model and a bootstrap evaluation of 1000 replicates using MEGA V6.0 [41].

### 2.5. Bacterial Isolation and Identification

*Escherichia coli* strains were isolated from fecal samples using screening on MacConkey agar (Solarbio, Beijing, China), as previously reported [42]. Subsequently, the isolated strains were confirmed to be *E. coli* using a PCR-based sequencing assay targeting the *16S rRNA* gene [43]. The *E. coli* strains of each sample were suspended in 20% glycerol and stored at −80 °C for further analysis.

### 2.6. Virulence Factor Determination of E. coli Strains

For each *E. coli* strain, a single colony of fresh bacterial culture on LB solid medium (Sangon Biotech, Shanghai, China) was selected and re-suspended in 150 µL ddH_2_O and then used as a DNA template in PCRs. Eight virulence genes for five pathotypes (EPEC, EHEC, ETEC, EAEC and EIEC) were detected in all the strains using two multiple PCRs, one for *aggR*, *lt* and *st*, and the other for *ipaH*, *eaeA*, *ehxA*, *stx1* and *stx2* [44,45,46].

### 2.7. Statistical Analysis

Differences in the positive rates of *Cryptosporidium* spp., *G. duodenalis*, *E. bieneusi* and *E. coli* in dairy goat kids with diarrhea among locations and age groups were analyzed using the χ^2^ test in SPSS V18.0 (IBM, Armonk, NY, USA). Significant differences were identified if the *p* value was less than 0.05.

### 2.8. Nucleotide Sequence Accession Numbers

Representative nucleotide sequences generated in this study are available in GenBank^TM^ with the accession numbers of OR220596-OR220604 for *E. bieneusi*, OR229417-OR229423 and OR232183-OR232196 for *Cryptosporidium* spp., OR230698-OR230699 for *E. coli* and OR237889-OR237906 for *G. duodenalis*.

## 3. Results

### 3.1. Occurrence of Cryptosporidium Species and Subtypes in Dairy Goat Kids with Diarrhea

Of 202 fecal samples detected in the present study, 76 (37.6%) were positive for *Cryptosporidium* spp. in dairy goat kids with diarrhea (Table 1). A significant difference among the positive rates of *Cryptosporidium* was confirmed among the five locations (χ^2^ = 31.602; *df* = 4; *p* < 0.001), with the highest in Fuping (50.0%, 65/130), followed by Yangling (40.0%, 8/20), Yanliang (10.5%, 2/19), Jingyang (10.0%, 1/10) and Sanyuan (0%, 0/23). Significant differences in the positive rates of *Cryptosporidium* were also found among three farms in Fuping (χ^2^ = 6.333; *df* = 2; *p* = 0.042). Additionally, a significant difference in positive rates was also identified among three age groups (χ^2^ = 29.520; *df* = 2; *p* < 0.001), with the highest in goat kids aged 2–4 weeks (52.0%, 53/102), followed by <2 weeks (40.4%, 19/47) and 4–12 weeks (7.5%, 4/53) (Table 1).

**Figure 2:**
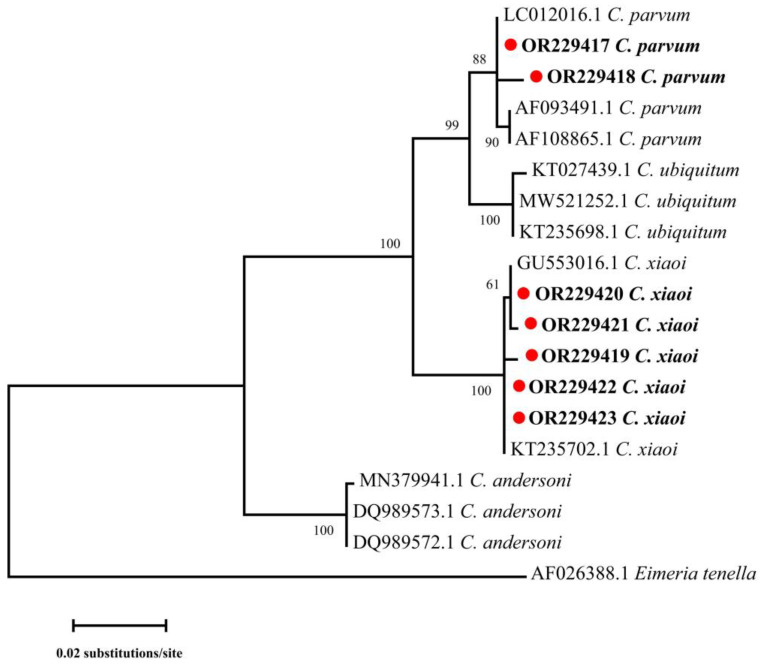
Phylogenetic relationships of *Cryptosporidium* spp. from sheep and goats based on the *SSU rRNA* gene by maximum likelihood analysis using general time-reversible model. Red-filled circles before the bold sample names represent species identified in the present study. Bootstrap values over 50 are presented at the nodes. *Eimeria tenella* (AF026388.1) is used as the outgroup. Representative sequences of each sequence type in this study are included in the phylogenetic analysis.

A sequence analysis of the *SSU rRNA* gene indicated the existence of two *Cryptosporidium* species in goat kids in the present study, namely *C. parvum* (*n* = 41) and *C. xiaoi* (*n* = 35) (Table 1 and Figure 2). Both *C. parvum* and *C. xiaoi* were found in goat kids in Fuping and Yangling, while only one species was recognized in Yanliang (*C. parvum*) and Jingyang (*C. xiaoi*). Meanwhile, both *C. parvum* and *C. xiaoi* were found in goat kids in all three age groups (Table 1).

Further sequence analysis based on the *gp60* gene showed subtype diversity within *C. parvum* and *C. xiaoi* in goat kids in the present study. As for *C. parvum*, 38 positive samples were successfully subtyped, with IIdA19G1 (*n* = 37) being the dominant one, followed by IIdA19G2 (*n* = 1). Meanwhile, two subtypes, namely XXIIIa (*n* = 22) and XXIIIg (*n* = 9), were found in 35 *C. xiaoi*-positive samples, with the mixed infection of XXIIIa and XXIIIg in two samples.

### 3.2. Occurrence of G. duodenalis Genotypes in Dairy Goat Kids with Diarrhea

**Figure 3:**
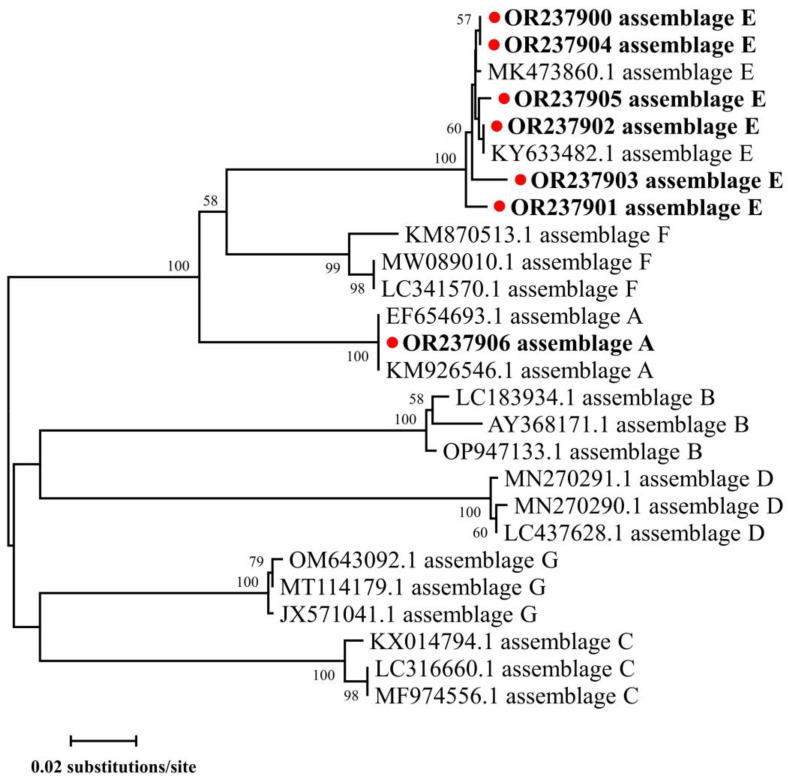
Phylogenetic relationships of *Giardia duodenalis* based on the *tpi* gene by maximum likelihood analysis using general time-reversible model. Red-filled circles before the bold sample names represent assemblages identified in the present study. Bootstrap values over 50 are presented at the nodes. Representative sequences of each sequence type in this study are included in the phylogenetic analysis.

The PCR analysis indicated the occurrence of *G. duodenalis* in 16.3% (33/202) of fecal samples, with the positive rates of 16.3% (33/202), 7.9% (16/202) and 14.9% (30/202) at the gene loci *gdh*, *tpi* and *bg*, respectively (Table 2). A significant difference in the positive rates of *G. duodenalis* was found among five locations (χ^2^ = 20.064; *df* = 4; *p* < 0.001) and three age groups (χ^2^ = 20.093; *df* = 2; *p* < 0.001), with the highest positive rates in goat kids from Jingyang (60.0%, 6/10) and aged 4–12 weeks (35.8%, 19/53). Meanwhile, a significant difference in the positive rates of *G. duodenalis* was also identified among three farms in Fuping (χ^2^ = 11.555; *df* = 2; *p* = 0.003). The sequence alignment of the gene loci *gdh*, *tpi* and *bg* and the phylogenetic analysis of the *tpi* gene locus indicated assemblages E and A in those samples positive for *G. duodenalis* (Table 2 and Figure 3). The most commonly identified genotype was assemblage E (*n* = 28), followed by assemblage A (*n* = 5). Both assemblages E and A were found in goat kids in Fuping, while only assemblage E was found in the other four regions. Meanwhile, both assemblages E and A were found in goat kids aged both <2 and 2–4 weeks, but only assemblage E was found in animals aged 4–12 weeks.

### 3.3. Occurrence of E. bieneusi Genotypes in Dairy Goat Kids with Diarrhea

Of the 202 fecal samples examined in the present study, 112 (55.4%) samples were positive for *E. bieneusi* in dairy goat kids with diarrhea (Table 3). The highest positive rate was found in goat kids from Yanliang (68.4%, 13/19), followed by Fuping (56.9%, 74/130), Sanyuan (56.5%, 13/23), Yangling (50.0%, 10/20) and Jingyang (20%, 2/10). Meanwhile, the positive rates were 60.8% (62/102), 54.7% (29/53) and 44.7% (21/47) in animals aged 2–4 weeks, 4–12 weeks and <2 weeks, respectively. Although the positive rates of *E. bieneusi* varied among locations (χ^2^ = 6.747; *df* = 4; *p* = 0.150) and age groups (χ^2^ = 3.393; *df* = 2; *p* = 0.183), no significant differences were found. Additionally, a significant difference in the positive rates of *E. bieneusi* was identified among three farms in Fuping (χ^2^ = 8.470; *df* = 2; *p* = 0.014).

A sequence analysis of the ITS gene of *E. bieneusi* identified nine known genotypes in the 112 sequences in the present study, namely CHG3, CHG1, BEB6, CHG5, CHG2, SX1, CHG28, COS-II and CD6 (Table 3). Among these identified genotypes, CHG3 was the most common genotype found in 51.8% (58/112) of goat kids, followed by CHG1 (25.0%, 28/112), BEB6 (7.1%, 8/112), CHG5 (6.3%, 7/112), CHG2 (3.6%, 4/112), SX1 (3.6%, 4/112), CHG28 (0.9%, 1/112), COS-II (0.9%, 1/112) and CD6 (0.9%, 1/112). Differences in genotype diversity were found among three locations, with nine (CHG3, CHG1, BEB6, CHG2, CHG5, SX1, CHG28, COS-II and CD6), four (CHG3, CHG5, SX1 and CHG2), three (CHG1, BEB6 and CHG3), two (CHG3 and CHG5) and two (CHG3 and BEB6) genotypes in Fuping, Yanliang, Yangling, Jingyang and Sanyuan, respectively. Meanwhile, seven (CHG1, CHG3, CHG5, CHG28, COS-II, SX1 and BEB6), seven (CHG3, CHG1, BEB6, CHG2, SX1, CHG5 and CD6) and four (CHG3, CHG1, CHG5 and BEB6) genotypes were identified in animals aged <2 weeks, 2–4 weeks and 4–12 weeks, respectively.

**Figure 4:**
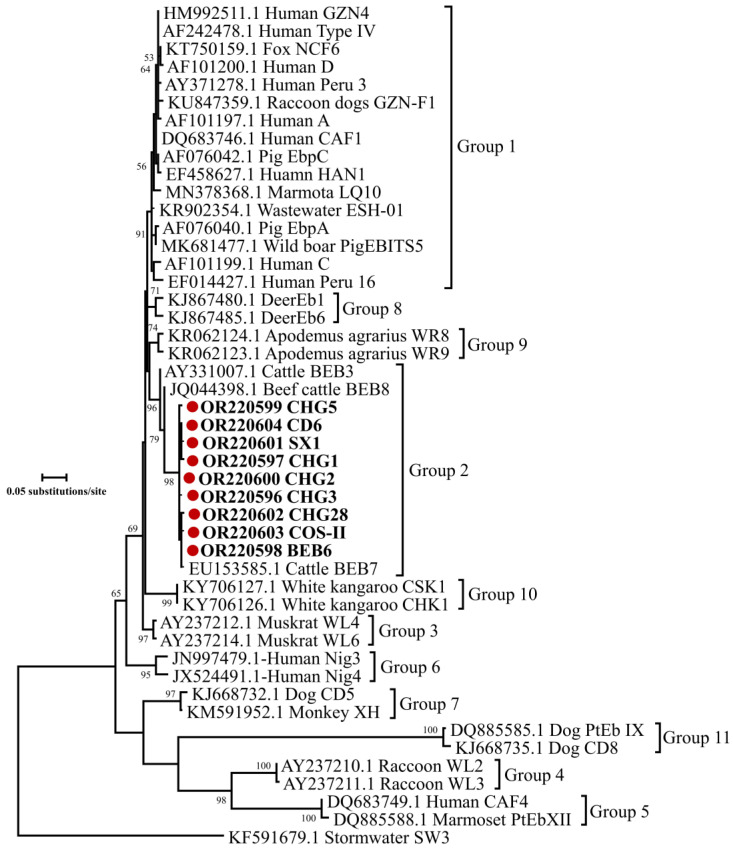
Phylogenetic relationships of representative sequences for the ITS genotypes of *E. bieneusi* identified in this study with reference sequences by maximum likelihood analysis using general time-reversible model. Red-filled circles before the bold sample names represent genotypes identified in the present study. Bootstrap values over 50 are presented at the nodes. Genotype SW3 from stormwater (KF591679.1) is used as the outgroup.

Further phylogenetic analysis based on the ITS gene of *E. bieneusi* showed that all nine genotypes (CHG3, CHG1, BEB6, CHG2, CHG5, SX1, CHG28, COS-II and CD6) belonged to the group 2, with increasing zoonotic potential (Figure 4). Since there were no variations among the sequences of each genotype, one representative sequence of each genotype was included in the phylogenetic analysis in Figure 4.

### 3.4. Occurrence of E. coli Pathotypes in Dairy Goat Kids with Diarrhea

The PCR-based sequencing analysis targeting the *16S rRNA* gene indicated that 78.7% (159/202) of fecal samples were positive for *E. coli* (Table 4). Although the positive rates of *E. coli* varied among locations (χ^2^ = 5.499; *df* = 4; *p* = 0.240) and age groups (χ^2^ = 0.164; *df* = 2; *p* = 0.921), no significant differences were identified. In addition, no significant differences in the positive rates of *E. coli* were identified among three farms in Fuping (χ^2^ = 4.214; *df* = 2; *p* = 0.122). To further understand the pathotypes of *E. coli* in the present study, a total of eight virulence factors (*eae*, *ehxA*, *stx2*, *stx1*, *lt*, *st*, *aggR* and *ipaH*) representing EPEC, EHEC, ETEC, EAEC and EIEC *E. coli* were examined (Table 4). Four of eight virulence genes were identified in 159 strains in this study, namely *eae* (66.7%, 106/159), *ehxA* (36.5%, 58/159), *stx2* (11.9%, 19/159) and *stx1* (9.4%, 15/159). However, no strains were positive for the gene loci *lt*, *st*, *aggR* and *ipaH* (Table 4). Meanwhile, most strains carried anywhere from one to four virulence genes of EPEC and EHEC.

### 3.5. Co-Infection of Pathogens for Cryptosporidium spp., G. duodenalis, E. bieneusi and E. coli in Dairy Goat Kids with Diarrhea

**Figure 5:**
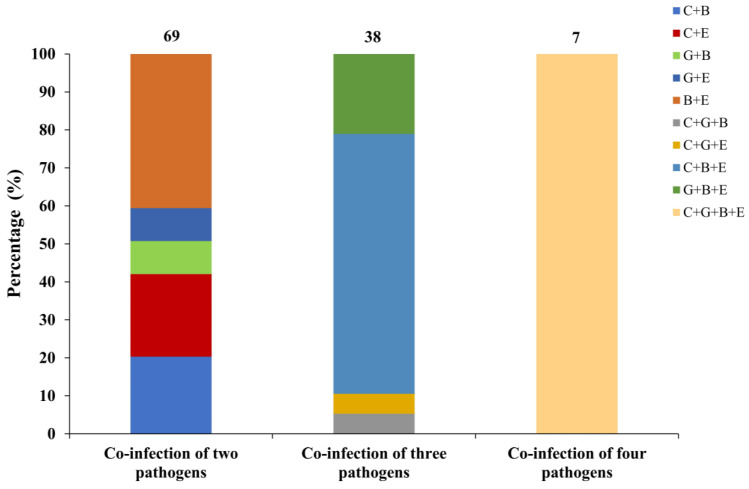
Percentage (%) of infection types within co-infections of two, three and four pathogens. C+B/C+E/G+B/G+E/B+E/C+G+B/C+G+E/C+B+E/G+B+E/C+G+B+E represent co-infection of *Cryptosporidium* spp. and *E. bieneusi*/*Cryptosporidium* spp. and *E. coli*/*G. duodenalis* and *E. bieneusi*/*G. duodenalis* and *E. coli*/*E. bieneusi* and *E. coli*/*Cryptosporidium* spp., *G. duodenalis* and *E. bieneusi*/*Cryptosporidium* spp., *G. duodenalis* and *E. coli*/*Cryptosporidium* spp., *E. bieneusi* and *E. coli*/*G. duodenalis*, *E. bieneusi* and *E. coli*/*Cryptosporidium* spp., *G. duodenalis*, *E. bieneusi* and *E. coli*, respectively. Numbers above bars denote sample size (*n*).

Co-infection of pathogens was found in 114 of 202 (56.4%) fecal samples, with 69, 38 and 7 samples positive for two, three and four pathogens, respectively (Figure 5). As for co-infection of two pathogens, a total of five types were identified, with the co-infection of *E. bieneusi* and *E. coli* being the most common one. As for co-infection of three pathogens, four types were found, with the co-infection of *Cryptosporidium* spp., *E. bieneusi* and *E. coli* being the dominant one.

## 4. Discussion

*Cryptosporidium* spp., *G. duodenalis*, *E. bieneusi* and *E. coli* are common and important zoonotic diarrheal pathogens greatly endangering the health of humans and animals, especially children and young animals [1,2,3,4]. The present study investigated the colonization frequency and genetic composition of *Cryptosporidium* spp., *G. duodenalis*, *E. bieneusi* and *E. coli* in diarrheal kids of Guanzhong dairy goats, which could expand our knowledge on the occurrence and distribution of these four pathogens in goats.

*Cryptosporidium* spp. are commonly found in sheep and goats in China. In the present study, the positive rate of *Cryptosporidium* spp. was 37.6% (76/202) in diarrheal kids of Guanzhong dairy goats from Shaanxi, which is similar to that seen in meat-producing goats (34.0%, 49/144), but higher than that in Saanen dairy goats (14.5%, 25/170) and northern Shaanxi white cashmere goats (9.5%, 30/315) in Shaanxi [32]. In addition, higher positive rates of *Cryptosporidium* spp. have been reported in sheep and goats in Ningxia (45.5%, 55/121), Henan (55.4%, 92/166) and Anhui (66.9%, 87/130) [47,48], while lower positive rates have been found in other provinces in China [49], such as Chongqing (5.3%, 16/301) [50], Heilongjiang (0.4%, 2/489) [51], Gansu (4.5%, 8/177) [52] and Xinjiang (0.9%, 3/318) [53]. The differences in the positive rates of *Cryptosporidium* spp. in sheep and goats are possibly caused by discrepancies in animal species and breeds, detection methods, sampling sizes, geographic regions and management practices.

*Cryptosporidium* infection in dairy goat kids was related to the age. In the present study, *Cryptosporidium* spp. were frequently found in all age groups, with the positive rates of 40.4% (19/47), 52.0% (53/102) and 7.5% (4/53) for goat kids aged <2, 2–4 and 4–12 weeks, respectively, indicating lower positive rates of animals aged >4 weeks compared with <4 weeks. Similar results have also been reported in sheep in Qinghai [54] and goats in Henan, Anhui and Qinghai [47], reflecting that immunity to *Cryptosporidium* in sheep and goats possibly increases with age. However, contrary results have been found in ewes and lambs in Inner Mongolia [55] and sheep in Henan, Anhui and Qinghai [47], reflected by higher positive rates in older animals compared with younger ones.

The sequences analysis indicated two *Cryptosporidium* species (*C. parvum* and *C. xiaoi*) in goat kids. *Cryptosporidium parvum* is a zoonotic species with a wide host range, including humans, cattle, sheep and goats, dogs, cats and mice [20]. Although *C. xiaoi* is the most common species in sheep and goats in previous reports [20,32,55], the present study found that *C. parvum* contributed to over half of the cases, indicating that potentially zoonotic strains of *Cryptosporidium* circulated on the investigated farms. A further subtyping analysis showed the existence of subtypes IIdA19G1 and IIdA19G2 in *C. parvum*-positive samples, with the former being the dominant one, which was also found in previous studies [56]. IIdA19G1 has also previously been reported in humans and animals [57,58,59,60], reflecting potential zoonotic transmission between humans and animals of this subtype. *Cryptosporidium xiaoi* was the other common species in the present study, which has also been widely reported in sheep and goats [31,32,53,54,56]. A further subtyping analysis based on the *gp60* gene locus found two potential goat-adapted subtypes (XXIIIa and XXIIIg) in *C. xiaoi*-positive samples, which was in accordance with a previous report [36].

Assemblages E and A were two *G. duodenalis* genotypes in goat kid samples in the present study, with the former being the major one, which is similar to studies in sheep and goats from Henan, Heilongjiang, Yunnan, Shaanxi and Qinghai [32,61,62,63,64,65]. Assemblage E has been widely reported in artiodactyls, such as cattle, sheep, pigs, goats and alpacas [24,66]. For a period of time, assemblage E was recognized as one animal-adapted genotype, but a report of this genotype in humans indicated its zoonotic potential [67]. Assemblage A is a zoonotic genotype found in humans, livestock, cats, dogs, beavers, guinea pigs and other primates and has also frequently been reported in sheep and goats [61,62,68,69].

A sequence analysis based on the ITS gene locus of 112 *E. bieneusi* isolates revealed nine known genotypes (CHG3, CHG1, BEB6, CHG5, CHG2, SX1, CHG28, COS-II and CD6) in goat kids in the present study, and these genotypes have been previously reported in sheep and goats [32,70,71,72]. Among the identified genotypes in this study, CHG3 was the most common genotype, which was also found in goats from Henan, Yunnan, Anhui, Chongqing and Shaanxi, as well as in sheep in Henan [71]. BEB6 is a zoonotic genotype with a wide host range, including humans [26], non-human primates [73], bovines [74], deer [75], takins [76], alpacas [77], cats [78] and birds [79]. Further phylogenetic analysis revealed that all nine genotypes belonged to the group 2. Although genotypes in the group 2 were reported to be ruminant-adapted at the beginning, more and more zoonotic genotypes identified in this group reflect an increasing risk of causing zoonotic infection between humans and animals [25,80].

A high positive rate of *E. coli* was commonly found in the present study, which is in accordance with previous reports in goats [42,81]. Further pathotype analysis based on the eight virulence genes of these *E. coli* strains indicated the existence of EPEC and EHEC, with the former being the dominant one in goat kids, while no strains were identified to be positive for virulence genes of ETEC, EAEC and EIEC. However, divergent pathotypes were reported in sheep, reflected by the dominance of EAEC and EHEC [42].

Co-infection of these diarrhea-related pathogens was found in goats in the present study, which has also been reported in sheep and goats in previous studies [32,52,53]. Previous studies have reported the co-infections of two pathogens (*Cryptosporidium* spp. and *G. duodenalis*, *G. duodenalis* and *E. bieneusi*, and *Cryptosporidium* spp. and *E. bieneusi*) and three pathogens (*Cryptosporidium* spp., *G. duodenalis* and *E. bieneusi*) in sheep and goats [32,52,53]. Although the present study did not find the co-infection of *Cryptosporidium* spp. and *G. duodenalis*, the co-infection of *E. coli* with *Cryptosporidium* spp., *G. duodenalis* and *E. bieneusi* was identified for the first time in sheep and goats.

## 5. Conclusions

This study explored the colonization frequency and genetic make-up of *Cryptosporidium* spp., *G. duodenalis*, *E. bieneusi* and *E. coli* in diarrheal kids of Guanzhong dairy goats from partial regions of Shaanxi Province. The findings in the present study indicated high positive rates and zoonotic species/genotypes/subtypes/pathotypes of these four diarrhea-related pathogens in goat kids. Considering the zoonotic potential of *Cryptosporidium* spp., *G. duodenalis*, *E. bieneusi* and *E. coli* in goat kids in this study, interventions are needed to prevent the cross-transmission of these diarrhea-related pathogens between animals and humans.

## Figures and Tables

**Table 1:** Occurrence and distribution of *Cryptosporidium* species and subtypes in dairy goat kids with diarrhea in partial regions of Shaanxi Province.

Factor		No. Examined	No. Positive (%)	Species (No.)	Subtype (No.)
Location					
Fuping	Farm 1	54	23 (42.6)	*C. parvum* (18) *C. xiaoi* (5)	IIdA19G1 (16) XXIIIg (4), XXIIIa (1)
	Farm 2	46	21 (45.7)	*C. parvum* (14) *C. xiaoi* (7)	IIdA19G1 (13) XXIIIg (3), XXIIIa (3)
	Farm 3	30	21 (70.0)	*C. xiaoi* (21)	XXIIIg (1), XXIIIa (18), XXIIIa + XXIIIg (2)
Sub-total		130	65 (50.0)	*C. parvum* (32) *C. xiaoi* (33)	IIdA19G1 (29) XXIIIa (22), XXIIIg (8), XXIIIa + XXIIIg (2)
Yanliang	Farm 4	19	2 (10.5)	*C. parvum* (2)	IIdA19G1 (2)
Yangling	Farm 5	20	8 (40.0)	*C. parvum* (7) *C. xiaoi* (1)	IIdA19G1 (6), IIdA19G2 (1) NA
Jingyang	Farm 6	10	1 (10)	*C. xiaoi* (1)	XXIIIg (1)
Sanyuan	Farm 7	23	0 (0)	–	–
Age (weeks)					
<2		47	19 (40.4)	*C. parvum* (14) *C. xiaoi* (5)	IIdA19G1 (12), IIdA19G2 (1) XXIIIg (5)
2–4		102	53 (52.0)	*C. parvum* (24) *C. xiaoi* (29)	IIdA19G1 (22) XXIIIa (22), XXIIIg (4), XXIIIa + XXIIIg (2)
4–12		53	4 (7.5)	*C. parvum* (3) *C. xiaoi* (1)	IIdA19G1 (3) NA
Total		202	76 (37.6)	*C. parvum* (41) *C. xiaoi* (35)	IIdA19G1 (37), IIdA19G2 (1) XXIIIa (22), XXIIIg (9), XXIIIa + XXIIIg (2)

NA: not available.

**Table 2:** Occurrence of *Giardia duodenalis* assemblages in dairy goat kids with diarrhea in partial regions of Shaanxi Province.

Factor		No. Examined	No. Positive (%)			Assemblage (No.)		
			*gdh*	*tpi*	*bg*	*gdh*	*tpi*	*bg*
Location								
Fuping	Farm 1	54	5 (9.3)	3 (5.6)	4 (7.4)	A (2), E (3)	A (2), E (1)	A (1), E (3)
	Farm 2	46	1 (2.2)	0 (0)	1 (2.2)	A (1)	–	A (1)
	Farm 3	30	8 (26.7)	8 (26.7)	8 (26.7)	A (2), E (6)	A (2), E (6)	A (2), E (6)
Sub-total		130	14 (10.8)	11 (8.5)	13 (10.0)	E (9), A (5)	E (7), A (4)	E (9), A (4)
Yanliang	Farm 4	19	2 (10.5)	0 (0)	2 (10.5)	E (2)	–	E (2)
Yangling	Farm 5	20	5 (25.0)	5 (25.0)	5 (25.0)	E (5)	E (5)	E (5)
Jingyang	Farm 6	10	6 (60.0)	0 (0)	5 (50.0)	E (6)	–	E (5)
Sanyuan	Farm 7	23	6 (26.1)	0 (0)	5 (21.7)	E (6)	–	E (5)
Age (weeks)								
<2		47	5 (10.6)	3 (6.4)	4 (8.5)	E (3), A (2)	E (1), A (2)	E (3), A (1)
2–4		102	9 (8.8)	8 (7.8)	9 (8.8)	E (6), A (3)	E (6), A (2)	E (6), A (3)
4–12		53	19 (35.8)	5 (9.4)	17 (32.1)	E (19)	E (5)	E (17)
Total		202	33 (16.3)	16 (7.9)	30 (14.9)	E (28), A (5)	E (12), A (4)	E (26), A (4)

**Table 3:** Occurrence of *Enterocytozoon bieneusi* genotypes in dairy goat kids with diarrhea in partial regions of Shaanxi Province.

Factor		No. Examined	No. Positive (%)	Genotype (No.)
Location				
Fuping	Farm 1	54	27 (50.0)	CHG1 (16), CHG2 (1), CHG3 (4), CHG28 (1), BEB6 (3), COS-II (1), SX1 (1)
	Farm 2	46	23 (50.0)	CHG1 (5), CHG2 (1), CHG3 (13), CHG5 (2), BEB6 (1), CD6 (1)
	Farm 3	30	24 (80.0)	CHG2 (1), CHG3 (20), BEB6 (1), SX1 (2)
Sub-total		130	74 (56.9)	CHG3 (37), CHG1(21), BEB6 (5), CHG2 (3), CHG5 (2), SX1 (3), CHG28 (1), COS-II (1), CD6 (1)
Yanliang	Farm 4	19	13 (68.4)	CHG3 (7), CHG5 (4), SX1 (1), CHG2 (1)
Yangling	Farm 5	20	10 (50.0)	CHG1 (7), BEB6(2), CHG3(1)
Jingyang	Farm 6	10	2 (20.0)	CHG3 (1), CHG5 (1)
Sanyuan	Farm 7	23	13 (56.5)	CHG3 (12), BEB6 (1)
Age (weeks)				
<2		47	21 (44.7)	CHG1 (13), CHG3 (3), CHG5 (1), CHG28 (1), COS-II (1), SX1 (1), BEB6 (1)
2–4		102	62 (60.8)	CHG3 (38), CHG1 (10), BEB6 (4), CHG2 (4), SX1 (3), CHG5 (2), CD6 (1)
4–12		53	29 (54.7)	CHG3 (17), CHG1 (5), CHG5 (4), BEB6 (3)
Total		202	112 (55.4)	CHG3 (58), CHG1 (28), BEB6 (8), CHG5 (7), CHG2 (4), SX1 (4), CHG28 (1), COS-II (1), CD6 (1)

**Table 4:** Occurrence of *Escherichia coli* pathotypes in dairy goat kids with diarrhea in partial regions of Shaanxi Province.

Factor		No. Examined	No. Positive (%)	Pathotype and Virulence Gene (No.)							
				EPEC		EHEC		ETEC		EAEC	EIEC
				*ehxA*	*eae*	*stx2*	*stx1*	*lt*	*st*	*aggR*	*ipaH*
Location											
Fuping											
	Farm 1	54	38 (70.4)	31	33	0	0	0	0	0	0
	Farm 2	46	35 (76.1)	5	16	2	6	0	0	0	0
	Farm 3	30	27 (90.0)	2	21	0	1	0	0	0	0
Sub-total		130	100 (76.9)	38	70	2	7	0	0	0	0
Yanliang	Farm 4	20	13 (65.0)	0	1	0	0	0	0	0	0
Yangling	Farm 5	19	17 (89.5)	9	10	6	2	0	0	0	0
Jingyang	Farm 6	10	9 (90.0)	4	9	0	2	0	0	0	0
Sanyuan	Farm 7	23	20 (87.0)	7	16	11	4	0	0	0	0
Age (weeks)											
<2		47	36 (76.6)	26	28	0	0	0	0	0	0
2–4		102	81 (79.4)	19	48	8	9	0	0	0	0
4–12		53	42 (79.2)	13	30	11	6	0	0	0	0
Total		202	159 (78.7)	58	106	19	15	0	0	0	0

## Data Availability

Data are contained within the article.
